# Vitamin D Status in Children With Forearm Fractures: Incidence and Risk Factors

**DOI:** 10.5435/JAAOSGlobal-D-20-00150

**Published:** 2020-08-20

**Authors:** Pooya Hosseinzadeh, Mahshid Mohseni, Arya Minaie, Gary M. Kiebzak

**Affiliations:** From the BHMG Pediatric Orthopedics (Dr. Hosseinzadeh), Baptist Children's Hospital, Miami, FL; the Division of Bone and Mineral Diseases (Dr. Mohseni), Department of Internal Medicine, and the Department of Orthopaedic Surgery (Mr. Minaie), Washington University in St. Louis, St. Louis, MO; and the Department of Orthopaedic Surgery (Dr. Kiebzak), Nemours Children's Hospital, Orlando, FL.

## Abstract

**Introduction:**

The association between vitamin D status and fracture characteristics in children remains ambiguous. We hypothesized that vitamin D deficient or insufficient children would have an increased risk of forearm fractures severe enough to require surgical management.

**Methods:**

One hundred children with low-energy forearm fractures were prospectively enrolled from a single hospital. Each participant answered a questionnaire focusing on the risk factors for vitamin D deficiency. Fractures were categorized as requiring nonsurgical or surgical management. Vitamin D status was based on the measurement of 25-hydroxyvitamin D (25(OH)D) concentration obtained during the clinic visit and compared between the two fracture groups.

**Results:**

The cohort exhibited a mean age of 9.8 ± 3.2 years (range: 3-15 years), comprising 65 (65%) men and 35 (35%) women. Overall, mean 25(OH)D was 27.5 ± 8.3 ng/mL. Using the Endocrine Society guidelines, 21% of patients were categorized as “vitamin D deficient” (25(OH)D ≤ 20 ng/mL) and 49% as “vitamin D insufficient” (25(OH)D: 21 to 29 ng/mL). Stratification by intervention revealed a mean 25(OH)D of 23.3 ± 8.8 ng/mL in the surgical group (n = 12) and 28.1 ± 8.1 in the nonsurgical group (n = 88) (*P* = 0.057). Fifty percent of the surgical group were “vitamin D deficient” compared with 17% of the nonsurgical group (*P* = 0.017). The relative risk of requiring surgical treatment in children with forearm fracture and vitamin D deficiency (25(OH)D < 20 ng/mL) was 3.8. 25(OH)D level, negatively correlated with body mass index (*r* = −0.21, *P* = 0.044); 9 surgical patients were overweight or obese (as defined by the criteria of the Centers for Disease Control and Prevention). 25(OH)D level was significantly lower in non-Caucasians compared with Caucasians (26.0 ± 7.2 versus 32.5 ± 9.9 ng/mL; *P* = 0.0008).

**Discussion:**

Vitamin D deficiency is common in children with forearm fractures and may be a contributing risk factor for forearm fractures requiring surgical management in children.

**Conclusion:**

Vitamin D deficiency and inefficiency are common in children with low energy forearm fractures, especially in obese children and in fractures requiring surgical treatment.

Fractures in children are common, with some estimates as high as 50% of boys and 40% of girls having at least one fracture by 18 years of age.^1-3^ The forearm is the most common fracture site in childhood, accounting for 25% of all pediatric fractures in the United States.^3-5^ The cost of treating radius fractures in the pediatric population of the United States has been cited to be more than $2 billion per year with an average cost of approximately $7,000 for treatment in an emergency department to nearly $24,000 for surgical treatment.^6,7^ Various risk factors have been identified or proposed including male sex, risk-taking behavior, poor nutrition, increased body mass index (BMI), low bone mineral density, poor bone quality, and lower socioeconomic status.^3,6,8,9,10,11,12^ Poor bone quality can lead to weakness in pediatric bones, making them susceptible to fractures. Similarly, low blood 25-hydroxyvitamin D (25(OH)D) levels, a marker for overall vitamin D status, can be a risk factor for subsequent fractures.^3,8,9,13^ Vitamin D deficiency and insufficiency are reported to be very common in children.^3,8,9^ Vitamin D deficiency leads to negative calcium balance, increasing parathyroid hormone in severe deficiencies and causing reabsorption of bone.^3^ The physiologic consequence is low bone mineral density and quality compromising bone strength. Patients can then become more susceptible to fractures at lower impact loads and potentially suffer greater fracture severity in the event of loading.

Establishing a relationship between this very common deficiency and the most common fracture in children is of notable healthcare and epidemiologic importance. However, to date, studies designed to investigate such a relationship between 25(OH)D and fracture risk have been inconclusive, with some studies showing a notable association and others refuting claims.^13,14,15,16,17^

Designing a population-based study to isolate the role of vitamin D status on fracture occurrence is difficult because it is necessary to control for the other major determinants of fractures, including mechanism. A different approach in attempting to establish a relationship between vitamin D status and pediatric fractures may instead be to assess vitamin D status in a cohort of children with forearm fractures and define patient characteristics associated with vitamin D deficiency. In this study, we prospectively studied a cohort of children with forearm fractures and assessed vitamin D status and factors associated with its deficiency.

## Methods

This was a prospective, nonrandomized study approved by our Institutional Review Board. Patients presenting to the clinic with children who sustained forearm fractures were approached regarding participation and informed consent for enrollment. Inclusion criteria included age of 3 to 17 years at the time of injury and a radius and/or ulna fracture. Exclusion criteria included high-impact traumatic fractures (such as those that occur in motor vehicle accidents), fractures in patients with adjacent bony cysts and neoplasm, and known metabolic bone disease. The primary outcome variable was blood concentration of 25(OH)D. Secondary and demographic variables included fracture characteristics, history of previous fractures, height and weight, BMI, obesity (defined by the Centers for Disease Control and Prevention age-adjusted BMI percentile), use of multivitamins, age, sex, and ethnicity. Fractures were classified as either requiring nonsurgical management (fractures not requiring reduction and those requiring closed reduction) or surgical management (fractures requiring surgical treatment including fixation with implant, usually percutaneous pinning or open reduction). All fractures were treated by one pediatric orthopaedic surgeon using strict criteria for surgical intervention. All closed fractures were treated initially by immobilization alone or closed manipulation and casting. Surgical intervention was exclusively used in patients with open fractures and in patients with closed fractures for which acceptable alignment was not either achieved by closed manipulation or could not be maintained because of loss of reduction in follow-up visits. The criteria recommended by Price^18^ were used for surgical the treatment of all patients in the study.

After the informed consent process and signing of the consent forms, the patient history relevant to the study was collected using a study intake form. Blood was collected by pin prick, and 2 to 6 drops of blood were deposited on a spot card. (This visit constituted the only research data collection visit; details of subsequent need for surgical management were obtained by chart review.) Blood spot cards were air-dried for 2 to 3 hours and then mailed to the laboratory for assay (ZRT Laboratory, Beaverton, OR). The samples were assayed using a liquid chromatography-mass spectrometry method.^19^ (We note that vitamin D status does not change rapidly and markedly unless a large bolus pharmacologic dose of vitamin D is given. Thus, sampling patients for the assessment of vitamin D status is not especially time dependent after the fracture event, as long as it is done before any new treatment commences.)

For the purposes of this study, the Endocrine Society guidelines were used, and vitamin D deficiency was defined as 25(OH)D ≤ 20 ng/mL and vitamin D insufficiency was defined as 25(OH) D of 21 to 29 ng/mL.^20^

### Statistical Analyses

When starting this study, no previous publications were focused on correlating vitamin D status and fracture severity in the pediatric population. Therefore, we did not have critical data elements with which to attempt a power analysis. We proceeded with IRB approval to enroll 100 subjects.

Descriptive statistics were calculated (mean and standard deviation, range, and 95% confidence intervals) for all variables. The primary analyses were comparing mean 25(OH)D concentrations and incidence of vitamin D deficiency (25-(OH)D ≤ 20) between the two fracture categories (surgical and nonsurgical group) using unpaired Student *t*-tests. Secondarily, mean 25(OH)D was compared between obese versus nonobese subjects, subjects with previous fractures versus no fractures, multivitamin users versus no multivitamin users, girls versus boys, and ethnic groups using unpaired Student *t*-tests. Correlation analyses were used to assess the relationship between 25(OH)D concentration and age. The percentage of obese versus nonobese subjects, multivitamin users versus no multivitamin users, girls versus boys, and ethnic groups were compared in each fracture category using contingency testing (Fishers exact test). Statistical significance was set as *P* < 0.05.

## Results

Nearly all of the forearm fractures resulted from play activities such as falling off of a bike, falling while running, and playing sport. Most patients (88%) had fractures that required nonsurgical treatment and only 12% were treated surgically. The details of fracture type and patient demographics are shown in Table 1. Mean blood 25(OH)D tended to be lower (17%, *P* = 0.057) in the surgical management group compared with the nonsurgical management group (Table 1), but this did not reach statistical significance. A significantly higher percentage of patients in the surgical group were vitamin D deficient compared with the nonsurgical group (50% versus 17%; *P* = 0.017). Patients requiring surgical management were significantly older and had greater BMI than patients not requiring surgical management (*P* = 0.0001) (Table 1). Seventy-five percent (9 of 12) of children in the surgical group were obese or overweight (after adjusting BMI for growth curves) compared with only 32% (29 of 82 who had BMI measured) of children in the group treated nonsurgically (*P* = 0.012).

**Table 1:** Vitamin D Status in Children With Forearm Fractures: Nonsurgical Versus Surgical Management

Variable	Nonsurgical Management			Surgical Management
	Group 1: No Reduction (n = 46)	Group 2: Closed Reduction (n = 42)	Group 1 + 2 (n = 88)	Surgical Reduction (n = 12)
Age, yr	9.66 ± 2.77	9.36 ± 3.64	**9.52** ± **3.2**	**12.07** ± 1.91*
Body mass index	19.1 ± 4.33	18.6 ± 3.71	**18.9** ± **4.0**	**23.9** ± 4.5**
25-hydroxyvitamin D, ng/mL	28.2 ± 8.25	28.0 ± 7.99	28.1 ± 8.08	23.3 ± 8.83

Bolded values designate significant (*P* < 0.05) differences (unpaired *t*-test; *P = 0.008, **P = 0.0001).

Notably, 70% of the cohort was insufficient/deficient using the Endocrine Society criteria (25(OH)D ≤ 30 ng/mL). Twenty-one patients in the cohort (both fracture groups) were vitamin D deficient (25(OH)D ≤ 20 ng/mL). These 21 patients were significantly older and had greater BMI than the group of patients who were vitamin D sufficient (25(OH)D ≥ 21 ng/mL) (Table 2). Being vitamin D deficient was associated with a greater risk of surgical management; 6 of 21 (28.6%) deficient patients were treated surgically compared with 6 of 79 (7.6%) patients without vitamin D deficiency, *P* = 0.0172 (Fishers exact test); the relative risk of requiring surgical treatment in children with low-energy forearm fractures and vitamin D deficiency was 3.8.

**Table 2:** Vitamin D Status in Children With Forearm Fractures: Patients With Vitamin D Deficiency 25(OH)D (≤20 ng/mL) Versus 25(OH)D (≥21 ng/mL)

Variable	Low (n = 21)	Normal (n = 79)	*P*
Age, yr	11.3 ± 2.20	9.42 ± 3.28	**0.013**
Body mass index	21.2 ± 3.623	19.0 ± 4.53	**0.0495**
25(OH)D, ng/mL	17.3 ± 2.45	30.2 ± 7.06	**<0.0001**
Surgical management	6 (28.6%)	6 (7.6%)	**0.0172**

25(OH)D = 25-hydroxyvitamin D

Bolded values designate statistically significant (*P* < 0.05) differences.

In this cohort, no difference was observed in vitamin D status between boys and girls, although 11 of 112 surgical patients were boys (Table 3). However, non-Caucasians (Hispanic and Black) had a mean 25(OH)D that was 20% lower than Caucasians (*P* = 0.0008) (Table 4). The proportion of non-Caucasians and Caucasians that were overweight or obese was not significantly different (30% [7 of 23] versus 42% [30 of 71]; *P* = 0.34).

**Table 3:** Vitamin D Status in Children With Forearm Fractures: Boys Versus Girls

Variable	Boys (n = 65)	Girls (n = 35)
Age, yr	**10.6** ± **3.2**	**8.32** ± **2.62**
Body mass index	20.0 ± 4.73	18.5 ± 3.67
Minutes in sun per day	103 ± 56.3	92.3 ± 53.8
25-hydroxyvitamin D, ng/mL	27.7 ± 7.15	27.2 ± 10.2

Bolded values designate statistically significant (*P* < 0.05) differences (unpaired *t*-test; *P* = 0.004).

**Table 4:** Vitamin D Status in Children With Forearm Fractures: Ethnic Groups

Variable	Black (n = 8)	Hispanic (n = 69)	All Non-Caucasian (n = 77)	Caucasian (n = 23)
Body mass index	19.8 ± 2.29	19.8 ± 4.4	19.8 ± 3.85	18.5 ± 4.92
25-hydroxyvitamin D, ng/mL	22.8 ± 6.18	26.4 ± 7.22	**26.0** ± **7.17**	**32.5** ± **3.89**

Bolded values designate statistically significant (*P* < 0.05) differences (unpaired *t*-test; *P* = 0.00008).

25(OH)D was found to be significantly correlated with age (*r* = −0.229, *P* = 0.0219) and BMI (*r* = −0.209, *P* = 0.0438).

## Discussion

This pilot study investigating the vitamin D status and factors associated with its deficiency in children with forearm fractures showed that patients treated surgically were typically non-Caucasian, overweight, or obese and had higher incidence of vitamin D deficiency. In fact, the relative risk of surgical management in children with vitamin D deficiency was 3.8. Our results corroborate the findings in a report by Minkowitz et al,^16^ showing that poor vitamin D status could affect fracture risk. Another important aspect of these results is that vitamin D insufficiency is common in children (21% vitamin D deficient and 49% vitamin D insufficient) and that being overweight/obese and non-Caucasian increases the likelihood of vitamin D deficiency.

To date, there is discordance in the literature regarding the role of poor vitamin D status and fracture risk with some studies showing that patients with fractures have lower vitamin than control subjects and others not showing this association.^13,14,15,16,17^ Only one other study that we are aware of has attempted to relate vitamin D status and facture severity in children. Minkowitz et al. recently reported the results of a study with 369 all-type fracture patients and 662 nonfracture control subjects aged 2 to 18 years. Although the occurrence of pediatric fractures was not associated with low 25(OH)D, it was found that low 25(OH)D was a notable independent risk factor for more severe fractures identified using a numerical global injury scoring system. As in our study, baseline differences in obesity were found between the study groups with fracture patients having a higher proportion of obese patients compared with control subjects without fractures. The incidence of vitamin D deficiency and insufficiency (21% vitamin D deficiency and 49% vitamin D insufficiency) in our study is similar to the reported incidence in the fracture group in the study by Minkowitz et al. (20% vitamin D deficiency and 45% vitamin D insufficiency).

It is not surprising to see the conflicting reports in the literature because there are many factors that can influence fracture occurrence and severity, which are difficult to control when designing a study to determine a causative role for low 25(OH)D. First, a bone fracture after minor trauma is the result of the interplay of many variables, including extrinsic factors which are the force and load characteristics of the fall itself and intrinsic factors which are the bone characteristics defining the patient's bone strength which influences the load-to-failure component. Genetic factors, illness, nutrition, medications, growth spurts (mineralization lagging behind the increase in bone size), balance, muscle mass, and strength all can potentially influence whether a fracture occurs after a fall. All the above-mentioned factors can potentially affect the severity of the fracture. Different bones have different composition of trabecular and cortical bones which affects the load to failure characteristics of each bone.

Our study is the first to define factors associated with vitamin D deficiency in children with forearm fractures showing a higher incidence of deficiency in the fractures requiring surgical treatment. The study, however, has limitations. One of the limitations is that we were not able to control for the force causing the injury. Other factors besides the characteristics of the initial fracture including the location of the fracture and the age of child play an important role in decision-making for surgical treatment. We tried to minimize this error by studying only one fracture type (forearm fractures), following a strict treatment protocol, and using strict criteria for surgical treatment.

Perhaps one of the main confounding factors in the interpretation of our results is the effect of obesity. It is hard to the separate effects of vitamin D deficiency and obesity because obesity is known to be associated with lower vitamin D levels.^6,10,11,16^ However, the relationship between obesity, bone strength, and low 25(OH)D is complex.^21-23^ Increased body weight is believed to lead to bigger and stronger bones, but there is likely a tipping point at which fat mass exceeds muscle mass and that could possibly negatively affect balance and coordination, which could lead to falls. Recent studies suggest that obesity in children and adolescence may actually lead to reduced bone mineral content below what would be expected based on weight, and for such patients, there is an increased risk of fracture.^20-22^ The mechanics of falling are also different in obese and nonobese individuals, which could be determinant in whether a fracture occurs. Clearly, the variables involved in who fractures and who doesn't are many and the patterns interrelationships are complex. Thus, it will be challenging if not impossible to design studies that can help reveal the primary determinant of fracture risk.

Our study has shown that vitamin D deficiency and insufficiency are common in children with low energy forearm fractures, and the deficiency is more common in obese children and children with fractures requiring surgical treatment. Owing to the high incidence of low vitamin D and the burden associated with forearm fractures in children, future large prospective studies are needed to further assess the effect this common nutritional deficiency in children.
